# Immune phenotype and histopathological growth pattern in patients with colorectal liver metastases

**DOI:** 10.1038/s41416-020-0812-z

**Published:** 2020-03-24

**Authors:** Stefan Stremitzer, Peter Vermeulen, Shannon Graver, Mark Kockx, Luc Dirix, Dongyun Yang, Wu Zhang, Judith Stift, Friedrich Wrba, Thomas Gruenberger, Heinz-Josef Lenz, Stefan J. Scherer

**Affiliations:** 1 USC/Norris Comprehensive Cancer Center, 1441 Eastlake Avenue, Los Angeles, CA 90033 USA; 2 Department of Surgery, Medical University Vienna, Waehringer Guertel 18-20, 1090 Vienna, Austria; 3 Histogenex, Sint-Bavostraat 78, 2610 Antwerp, Belgium; 4 University of Wuerzburg, Biocenter, Am Hubland, 97074 Wuerzburg, Germany; 5 Sint-Augustinus Hospital Oncology Center, Medical Oncology, Oosterveldlaan 24, 2610 Antwerp, Belgium; 6 Clinical Institute of Pathology, Medical University Vienna, Waehringer Guertel 18-20, 1090 Vienna, Austria; 7 Department of Surgery, Social Medical Center South, Kundratstrasse 3, 1100 Vienna, Austria

**Keywords:** Predictive markers, Prognostic markers, Colon cancer, Rectal cancer, Surgical oncology

## Abstract

**Background:**

Patients with desmoplastic (angiogenic) histopathological growth pattern (HGP) colorectal liver metastases (CLM) might derive more benefit from bevacizumab-based chemotherapy than those with replacement (non-angiogenic) HGP. This study investigated the association of HGP with the immune phenotype (IP) and clinical outcome after liver resection.

**Methods:**

CLM of patients treated with perioperative bevacizumab-based chemotherapy and liver resection were investigated. Association of HGP and IP with response, recurrence-free survival (RFS) and overall survival (OS) was investigated.

**Results:**

One hundred and eighteen patients (M/F 66/52, median age 62.3 (31.0–80.4) years, median follow-up 32.2 (5.0–92.7) months) were enrolled. The inflamed IP was associated with the desmoplastic HGP. The desmoplastic HGP was associated with better radiological and histological response compared to the replacement HGP, respectively. The replacement HGP was associated with shorter RFS (8.7 versus 16.3 months, HR 2.60, *P* = 0.001) and OS (36.6 months versus not reached, HR 2.32, *P* = 0.027), respectively. The non-inflamed IP was associated with shorter RFS (10.8 versus 16.5 months, HR 1.85, *P* = 0.029). The HGP but not the IP remained significant in multivariable analysis for RFS.

**Conclusions:**

The desmoplastic HGP is associated with the inflamed IP and HGP may be a potential biomarker for adjuvant treatment that includes targeting the immune contexture.

## Background

Liver resection is the gold standard for the treatment of resectable colorectal liver metastases (CLM) and associated with long-term survival.^1^ In combination with perioperative chemotherapy, liver resection offers the chance of cure in approximately 25% of the patients.^2^ The addition of the anti-vascular endothelial growth factor (anti-VEGF) antibody bevacizumab to combination chemotherapy targeting the angiogenesis pathway is associated with high radiological response and improved histological response rates.^3–5^ Biomarkers to predict the efficacy of bevacizumab-based chemotherapy in this setting are urgently needed.

The histopathological growth patterns (HGPs) of CLM, which are defined by the specific interface between the tumour and the surrounding normal liver, have been identified as a promising biomarker.^6,7^ Two common HGPs (desmoplastic and replacement) and one rare HGP (pushing) have been described and have been related to the means of vascularisation of liver metastases.^6^ In the desmoplastic HGP, the tumour is separated from the liver tissue by a rim of desmoplastic tissue, which contains new blood vessels as a result of angiogenesis. The pushing HGP is similar to the desmoplastic, but the desmoplastic rim is absent. In striking contrast to these HGPs, the replacement growth pattern shows cancer cells growing into the liver cell plates and replacing the hepatocytes. They respect the normal tissue architecture and use the blood vessels of the liver and the supporting stroma. In this HGP, tumour vascularisation is thus achieved by a non-angiogenic process termed vessel ‘co-option’, in which normal sinusoidal blood vessels are hijacked by the tumour. Vessel co-option appears to be a frequently overlooked mechanism of tumour vascularisation, also in other tumour types, for example brain tumours, lung tumours, primary liver tumours and skin and lymph node metastases.^8^

We and others have repeatedly shown that the HGPs have a prognostic and predictive value for patients with resected liver metastases not only from colorectal cancer but also from uveal melanoma.^7,9,10^ This underscores the distinct biology of the different growth patterns. The replacement growth pattern has been associated with poor pathological responses (large proportion of viable cancer) and poor morphological responses on computed tomographic (CT) imaging in patients who received chemotherapy and anti-VEGF treatment prior to surgery for colorectal cancer liver metastases.^9^ In the same study, the replacement growth pattern occurred more frequently in new liver lesions, which appeared during systemic treatment.^9^ Taken together, the replacement growth pattern predicts both intrinsic and acquired resistance to systemic treatment in patients with colorectal cancer liver metastases. Observations done in preclinical models seem to suggest that cancer cells of a tumour in the liver with a replacement HGP have undergone partial epithelial-to-mesenchymal transition, are more motile and have adopted stem cell-like characteristics, which could explain the more aggressive behaviour of the replacement HGP.^9,11^

Chen and Mellman have proposed a model in which intrinsic factors of a tumour, such as the cytokines produced by the cancer cells, combined with extrinsic factors, such as the microenvironment, in which the tumour is growing, determine the immune phenotype.^12^ Three distinct immune phenotypes have been proposed by the authors: the immune desert, the inflamed, and the immune-excluded phenotype. The immune phenotypes obviously reflect the hurdles that must be overcome for a patient to respond to immunotherapy but probably also influence, at least partly, the response to chemotherapy and radiotherapy. Some isolated published observations suggest that the HGPs of liver metastases are associated with distinct immune phenotypes.^6,13,14^ The aim of our study was to assess the HGPs of resected colorectal cancer liver metastases and compared these with the immune phenotypes and investigated their clinical value as predictive and/or prognostic biomarkers.

## Methods

Patients with resectable or borderline resectable CLM who underwent liver resection in curative intent after 3 months of neoadjuvant and adjuvant chemotherapy including bevacizumab were analysed (2005–2011). The last dose of bevacizumab was administered 5 weeks before the liver resection. Clinical data were obtained from a prospectively maintained database. The HGPs of CLM were assessed according to international consensus guidelines.^7^ Briefly, in every available haematoxylin–eosin-stained tissue sections of each liver metastasis, the entire interface between liver tissue and tumour tissue was evaluated. The relative fraction of each growth pattern that constitutes >5% of the total length of the interface was estimated. The dominant HGP was recorded for further analysis (>50% of the total length of the interface). Images of HGPs are given in Supplementary Figs. S1 and S2. The immune phenotypes were based on the distribution pattern of cytotoxic T lymphocytes in CD8-immunostained tissue sections as previously described.^12^ Briefly, the dominant immune phenotype was used for further analyses. The entire tissue section was evaluated at low power magnification. A ‘desert’ immune phenotype was identified when no or a minimal amount of CD8-positive immune cells were present around and in the metastasis. When a clear rim of CD8-positive immune cells surrounded the liver metastasis and only a minimal amount of CD8-positive immune cells was present within the metastasis, the excluded immune phenotype was identified. Whenever a distinct CD8-positive immune infiltrate was present in the metastasis, and not only at the tumour–liver interface, the immune phenotype was called ‘inflamed’. Images of immune phenotypes are given in Supplementary Figs. S3 and S4. Associations of immune phenotypes and HGPs with recurrence-free survival (RFS) and overall survival (OS) were analysed.

Radiological response was assessed according to RECIST 1.1.^15^ Only patients with partial response (PR) or disease stabilisation after neoadjuvant chemotherapy underwent liver resection (progressive disease rate <5%). Histological response was assessed as proposed by Rubbia-Brandt et al.^16^ Tumour regression grade (TRG) 1 and 2 were classified as major histological response (MjHR), TRG 3 as partial histological response (PHR) and TRG 4 and 5 as no histological response (NHR).

### Follow-up

Patients were reassessed with clinical investigation, contrast-enhanced CT and blood test including carcino‐embryonic antigen every 3 months for the first 2 years after the liver resection and every 6 months thereafter.

### Statistical analysis

Normally distributed variables were given as mean and standard deviation (s.d.) or otherwise as median and range. Continuous variables were compared using Student’s *T* test if normally distributed or otherwise using Mann–Whitney *U* test. Categorical variables were compared using chi-square test. Kaplan–Meier plots were drawn to compare RFS and OS after liver resection and compared using log-rank test. RFS and OS were defined as time from liver resection until recurrence and death, respectively, or censored at the time of the last follow-up. Univariable Cox regression analyses were performed for each of the influence factors to evaluate their association with RFS and OS. Statistically significant factors in univariable analysis were included in a multivariable analysis. *P* values <0.05 were considered to be statistically significant. All statistical analyses were performed using SPSS 24.0 (IBM, Armonk, NY, USA).

## Results

One hundred and eighteen patients were enrolled. The male/female ratio was 66 (56%)/52 (44%). The median age was 62.3 (31.0–80.4) years. The median follow-up was 32.2 (5.0–92.7) months. Of the 118 patients, 99 received oxaliplatin-based chemotherapy, 16 received irinotecan-based chemotherapy and 3 received both agents. One of the 106 (1%) patients had mismatch repair-deficient CLM. Patient demographics are given in Table 1.

**Table 1:** Patient demographics.

Parameter	Immune phenotype			HGP		
	Inflamed	Non-inflamed	*P* value	Angiogenic	Non-angiogenic	*P* value
Age (years)	62.3	61.5	0.70	61.6	62.4	0.73
Sex			0.38			0.82
Male	21	19		26	14	
Female	17	24		25	16	
Radiological response			0.39			0.018
PR	31	32		43	20	
SD	5	10		5	10	
Histological response			0.09			0.009
MjHR	19	11		25	5	
PHR	9	14		14	9	
NHR	10	17		12	15	
Primary tumour side			0.09			0.009
Right	14	8		19	3	
Left	24	34		31	27	
Primary tumour site			0.10			0.64
Rectum	10	19		17	12	
Colon	28	23		33	18	
Number of metastases			0.65			0.49
1–2	24	24		32	16	
>2	14	19		19	14	
Size of metastases			0.76			0.75
≤5 cm	33	36		44	25	
>5 cm	5	7		7	5	
Timing of metastases			1.00			0.24
Metachronous	13	15		15	13	
Synchronous	25	27		35	17	
Distribution of metastases			0.38			0.82
Unilobar	21	19		26	14	
Bilobar	17	24		25	16	
KRAS status			0.16			0.47
mt	10	19		16	13	
wt	25	23		31	17	
Mismatch repair			0.42			0.43
Proficient	29	41		60	45	
Deficient	1	0		0	1	

Forty-seven of the 118 (40%) patients harboured the replacement HGP, while 71 (60%) patients harboured the desmoplastic HGP. None of the patients harboured the pushing HGP. In 81 of the 118 (69%) patients, the immune phenotype of the CLM could be assessed. Thirty-eight of the 81 (47%) patients had an inflamed immune phenotype, while 43 (53%) patients had a non-inflamed immune phenotype (19 desert, 24 excluded). The inflamed immune phenotype was associated with the desmoplastic HGP (32 of 38 versus 19 of 43, odds ratio 6.74 (95% confidence interval 2.34–19.44), *P* < 0.001).

The desmoplastic HGP was associated with better radiological response compared to the replacement HGP (PR 43, stable disease (SD) 5 patients versus PR 20, SD 10 patients, *P* = 0.018). The desmoplastic HGP was also associated with better histological response compared to the replacement HGP (MjHR 25, PHR 14, NHR 12 patients versus MjHR 5, PHR 9, NHR 15 patients, *P* = 0.009).

**Fig. 1:**
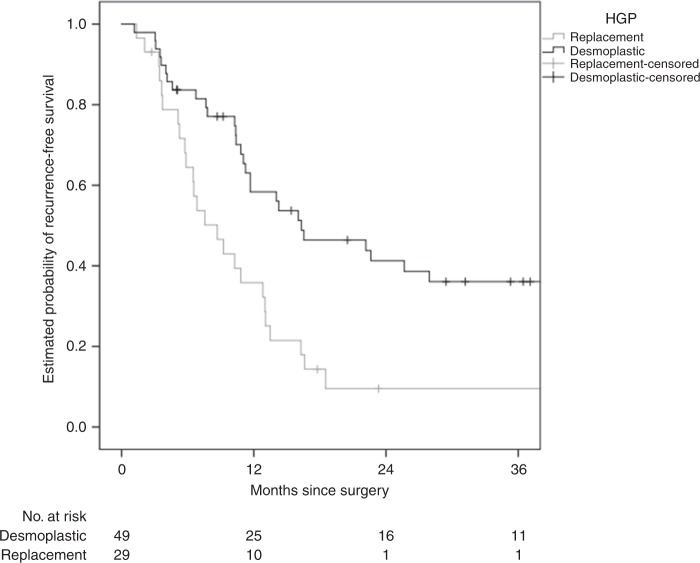
HGP and association with RFS. The replacement HGP is associated with shorter RFS than the desmoplastic HGP (median 8.7 versus 16.3 months, log-rank *P* < 0.001).

**Fig. 2:**
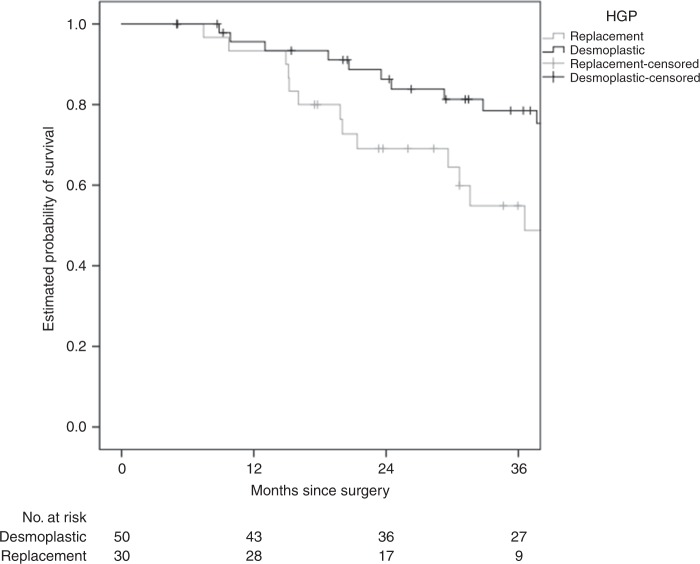
HGP and association with OS. The replacement HGP is associated with shorter OS than the desmoplastic HGP (median 36.6. months versus not reached, log-rank *P* = 0.023).

**Fig. 3:**
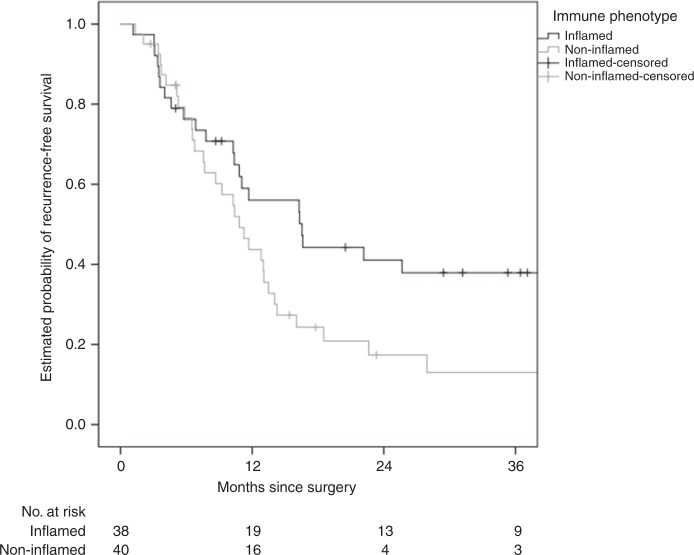
IP and association with RFS. The non-inflamed IP is associated with shorter RFS than the inflamed IP (median 10.8 versus 16.5 months, log-rank *P* = 0.026).

**Fig. 4:**
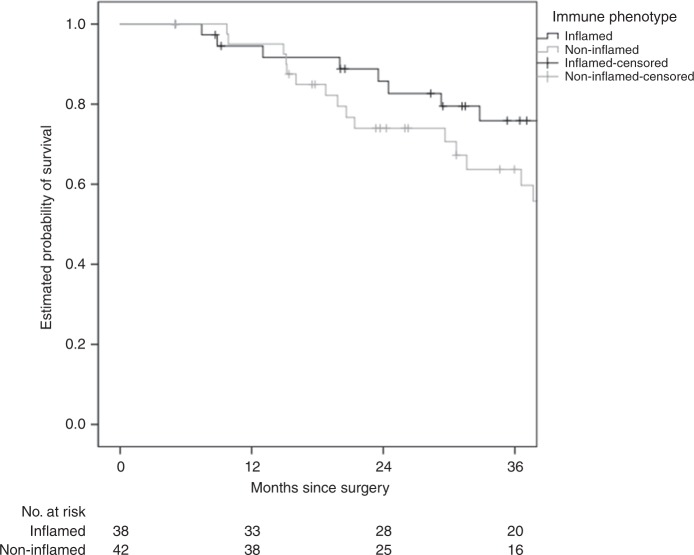
IP and association with OS. The non-inflamed IP is not associated with a statistically significant difference in OS compared to the inflamed IP (median 48.2 months versus not reached, log-rank *P* = 0.23).

The replacement HGP was associated with shorter RFS (median 8.7 versus 16.3 months, hazard ratio (HR) 2.60 (1.50–4.51), *P* = 0.001) and OS (median 36.6 months versus not reached, HR 2.32 (1.10–4.90), *P* = 0.027), respectively (Figs. 1 and 2). The non-inflamed immune phenotype was associated with shorter RFS (median 10.8 versus 16.5 months, HR 1.85 (1.07–3.21), *P* = 0.029) but not with OS (median 48.2 months versus not reached, HR 1.59 (0.74–3.40), *P* = 0.23) (Figs. 3 and 4).

For RFS, HGP, immune phenotype, histological response, KRAS status, distribution of metastases and number of metastases >2 were statistically significant in univariable analyses and were included in multivariable analyses. The HGP but not the immune phenotype remained significant in multivariable analysis for RFS (HR 2.84 (1.58–5.11), *P* = 0.001). Other factors associated with RFS in multivariable analysis were KRAS status and number of metastases >2 (Supplementary Table S1). For OS, HGP, age >70 years, radiological response, histological response, KRAS status, timing of metastases, distribution of metastases and number of metastases >2 were statistically significant in univariable analyses and were included in multivariable analyses. The HGP did not remain in multivariable analysis for OS. Factors associated with OS in multivariable analysis were radiological and histological response, KRAS status, timing of metastases and number of metastases >2 (Supplementary Table S2).

## Discussion

This study showed for the first time that the angiogenic desmoplastic HGP is associated with the inflamed immune phenotype and is associated with response and clinical outcome in patients with CLM who underwent bevacizumab-based neoadjuvant chemotherapy and liver resection. The results of this study support the concept that immune regulatory and angiogenesis pathways interact and that the HGP may serve as a predictive and/or prognostic biomarker.

In a previous landmark study, Frentzas et al. showed that the replacement HGP is associated with vessel co-option rather than angiogenesis. The authors demonstrated that this HGP is associated with poorer histological and morphological response and survival in patients who underwent liver resection after preoperative therapy with bevacizumab.^9^ In line with this previous study, the replacement HGP was associated with worse response and survival compared to the desmoplastic HGP in our study, which may be related to resistance to bevacizumab of these tumours due to the use of vessel co-option instead of angiogenesis.

The most striking finding in our study, however, was that the desmoplastic HGP was associated with the inflamed immune phenotype. To the best of our knowledge, this is the first study identifying this association of this angiogenic HGP and the inflamed immune phenotype in this setting. These results support the notion of previous studies that molecular pathways of angiogenesis and immune regulation interact. A post hoc analysis of the NSABP C-08 study investigating the clinical relevance of mismatch repair deficiency in patients with stage II/III colon cancer receiving adjuvant oxaliplatin-based chemotherapy+/−bevacizumab showed that in patients with mismatch repair-deficient tumours the addition of bevacizumab was associated with longer OS (HR 0.52, *P* = 0.03).^17^ In contrast to this finding, no difference between the treatment groups was observed in patients with mismatch repair-proficient tumours. These results suggest that in patients with immunogenic mismatch repair-deficient tumours bevacizumab may act as an immune modulator and may increase the host’s immune response. This interaction of inhibition of angiogenesis with immune response has also been shown in patients with renal cell cancer and melanoma, in whom the addition of bevacizumab to anti-programmed death ligand 1 (anti-PD-L1) or anti-CTLA4 therapy increased the T cell response.^18,19^

The recurrence rate after liver resection is high (approximately 75%), and new agents are urgently needed to improve outcome in a multi-disciplinary treatment approach.^2^ The identified link between HGP, response and immune pathways may help identifying new drug combination that may include e.g. immune checkpoint inhibitors in this setting. So far, efficacy of these new agents has only been shown in mismatch repair-deficient tumours, while resectable CLM are almost exclusively mismatch repair proficient.^20^ However, the microenvironment and tumour biology in patients with liver-limited, resectable CLM may be different compared to patients with multi-site metastases and immunotherapy may have an indication in this setting. New combination therapies are currently studied in patients with resectable CLM (e.g. ClinicalTrials.gov Identifier: NCT02754856), but their appropriate sequence also needs to be investigated in prospective clinical trials. Moreover, prospective studies are warranted to investigate whether a clinically active, inflamed immune phenotype can be induced by neoadjuvant treatment with bevacizumab. In addition, expression analyses of immune checkpoints, e.g. PD-L1, may help identifying subgroups of patients with mismatch repair-proficient tumours who may benefit from additional adjuvant immune checkpoint inhibition to reduce the recurrence rate and improve survival. A treatment effect of bevacizumab on the HGP and the immune phenotype as a form of immune modulation may also later guide the investigation of combination therapies together with immune checkpoint inhibitors in patients with multi-site, mismatch repair-proficient tumours. New combination therapies including immunotherapy and bevacizumab are currently investigated in this setting (e.g. ClinicalTrials.gov Identifier: NCT03555149).

With respect to future patient management, the association of HGP and immune phenotype may help identifying patients who benefit from immunotherapy with or without bevacizumab-based chemotherapy especially in the adjuvant setting.

Despite being a potential predictive and/or prognostic biomarker for additional immunotherapy, HGP may also guide selection of patient who do benefit from adjuvant bevacizumab-based therapy. Previous studies have shown that the desmoplastic HGP is associated with proliferating endothelial cells, immature and leaky blood vessels lacking pericyte cover and causing stromal fibrin deposition and vascular hot spots, which have shown to be an effect of VEGF.^6,21,22^ Moreover, it has been suggested that one of the effects of anti-VEGF-based combination therapy is the normalisation of tumour vessels to improve the delivery of chemotherapeutic agents.^23^ This is in keeping with our previous observation that variants of genes involved in angiopoietin and pericyte pathways are associated with clinical outcome after neoadjuvant bevacizumab-based chemotherapy and resection of CLM.^24^ The desmoplastic reaction of liver metastases has been shown to be related to ductular proliferation and growth factors secreted by cancer-associated fibroblasts such as the chemokine C-X-C chemokine motif ligand 12 and transforming growth factor beta that are also angiogenic are involved in this process.^25^ This interaction of pathways involved in both desmoplasia and angiogenesis explains the efficacy of bevacizumab in patients with CLM showing a desmoplastic HGP. Thus bevacizumab may be omitted in the adjuvant setting after liver resection in patients with resected CLM harbouring an HGP associated with resistance to anti-VEGF therapy. However, it has to be acknowledged that HGP and immune phenotype can currently only be assessed in resected CLM. This is a limitation of these potentially predictive biomarkers that would confine their use to adjuvant therapy.

A distinct HGP is primarily a result of the underlying tumour biology; however, pretreatment of CLM may influence HGPs. Frentzas et al. showed that the fraction of replacement HPG was significantly higher in treatment-resistant CLM that developed during systemic therapy.^9^ Interestingly, in the current study none of these uniformly bevacizumab-pretreated patients had predominately pushing HGP, which, similar to the replacement HGP, has previously been reported to be associated with poor clinical outcome.^22^ However, future studies are need to elucidate this matter.

A strength of this study is the uniform treatment of patients with neoadjuvant combination chemotherapy including bevacizumab. However, some limitations of this study have to be acknowledged. One limitation is the retrospective design and another limitation is the limited patient number. Moreover, both the HGP and the immune phenotype could be assessed in only 69% of the patients.

This study showed for the first time that the desmoplastic HGP is associated with the inflamed immune phenotype suggesting that angiogenesis and immune pathways interact. The HGP may therefore serve as predictive and/or prognostic biomarker in patients who undergo liver resection after neoadjuvant bevacizumab-based chemotherapy.

## Supplementary information

Supplementary Files

## Data Availability

The data sets generated and/or analysed during the current study are not publicly available due to it containing patients’ personal data, but anonymised data are available from the corresponding author on reasonable request.
