# Supplementary material for: Immune phenotype and histopathological growth pattern in patients with colorectal liver metastases
Source: Br J Cancer. 2020 Mar 24;122(10):1518–24. doi: 10.1038/s41416-020-0812-z (PMC7217855; doi:10.1038/s41416-020-0812-z)

Supplementary Table S1. Univariable and multivariable Cox regression analyses of factors associated with RFS (not applicable, n.a.)

| **Variable** | **Univariable** | | **Multivariable** | |
| --- | --- | --- | --- | --- |
|  | **HR (95% CI)** | ***P*-value** | **HR (95% CI)** | ***P*-value** |
| Histopathological Growth Pattern  Replacement  Desmoplastic | 2.60 (1.50, 4.51)  1 (Reference) | 0.001 | 2.84 (1.58, 5.11)  1 (Reference) | 0.001 |
| Immune phenotype  Non-inflamed  Inflamed | 1.85 (1.07, 3.21)  1 (Reference) | 0.029 | n.a. | 0.32 |
| Sex  Female  Male | 0.91 (0.53, 1.56)  1 (Reference) | 0.73 |  |  |
| Age  >70 years  ≤70 years | 0.82 (0.41, 1.64)  1 (Reference) | 0.58 |  |  |
| Radiological response  PR  SD | 0.58 (0.31, 1.09)  1 (Reference) | 0.09 |  |  |
| Histological response  MjHR  PHR  NHR | 0.42 (0.21, 0.83)  0.44 (0.24, 0.83)  1 (Reference) | 0.012 | n.a. | 0.37 |
| KRAS  Mutant  Wild-type | 2.48 (1.41, 4.34)  1 (Reference) | 0.002 | 2.73 (1.51, 4.92)  1 (Reference). | 0.001 |
| Primary tumor side  Right  Left | 0.59 (0.30, 1.15)  1 (Reference) | 0.12 |  |  |
| Primary tumor site  Colon  Rectum | 0.93 (0.54, 1.61)  1 (Reference) | 0.80 |  |  |
| Timing of metastasis  Metachronous metastases  Synchronous metastases | 1.09 (0.62, 1.90)  1 (Reference) | 0.77 |  |  |
| Distribution of metastases  Bilobar  Unilobar | 1.94 (1.13, 3.35)  1 (Reference) | 0.017 | n.a | 0.40 |
| Number of metastases  >2  1-2 | 2.08 (1.20, 3.60)  1 (Reference) | 0.009 | 2.86 (1.55, 5.27)  1 (Reference) | 0.001 |
| Size of metastases  >5cm  ≤5cm | 1.21 (0.59, 2.47)  1 (Reference) | 0.61 |  |  |

Supplementary Table S2. Univariable and multivariable Cox regression analyses of factors associated with OS (not applicable, n.a.)

| **Variable** | **Univariable** | | **Multivariable** | |
| --- | --- | --- | --- | --- |
|  | **HR (95% CI)** | ***P*-value** | **HR (95% CI)** | ***P*-value** |
| Histopathological Growth Pattern  Replacement  Desmoplastic | 2.32 (1.10, 4.90)  1 (Reference) | 0.027 | n.a. | 0.84 |
| Immune phenotype  Non-inflamed  Inflamed | 1.59 (0.74, 3.40)  1 (Reference) | 0.23 |  |  |
| Sex  Female  Male | 0.84 (0.40, 1.77)  1 (Reference) | 0.65 |  |  |
| Age  >70 years  ≤70 years | 2.40 (1.04, 5.53)  1 (Reference) | 0.039 | n.a. | 0.72 |
| Radiological response  PR  SD | 0.40 (0.18, 0.91)  1 (Reference) | 0.029 | 0.35 (0.14, 0.89)  1 (Reference) | 0.028 |
| Histological response  MjHR  PHR  NHR | 0.33 (0.14, 0.79)  0.34 (0.12, 0.95)  1 (Reference) | 0.018 | 0.48 (0.17, 1.35)  0.26 (0.08, 0.78)  1 (Reference) | 0.047 |
| KRAS  Mutant  Wild-type | 2.85 (1.35, 6.03)  1 (Reference) | 0.006 | 2.25 (0.98, 5.15)  1 (Reference) | 0.056 |
| Primary tumor side  Right  Left | 0.72 (0.27, 1.91)  1 (Reference) | 0.51 |  |  |
| Primary tumor site  Colon  Rectum | 1.05 (0.49, 2.24)  1 (Reference) | 0.91 |  |  |
| Timing of metastasis  Metachronous metastases  Synchronous metastases | 2.41 (1.14, 5.09)  1 (Reference) | 0.021 | 2.70 (1.16, 6.30)  1 (Reference) | 0.022 |
| Distribution of metastases  Bilobar  Unilobar | 2.83 (1.28, 6.29)  1 (Reference) | 0.010 | n.a. | 0.50 |
| Number of metastases  >2  1-2 | 2.14 (1.01, 4.51)  1 (Reference) | 0.046 | 2.70 (1.05, 6.94)  1 (Reference) | 0.039 |
| Size of metastases  >5cm  ≤5cm | 0.58 (0.18, 1.93)  1 (Reference) | 0.38 |  |  |

Supplementary Figure S1. Desmoplastic HGP (fibrous rim (middle part of image) separates liver from cancer tissue).


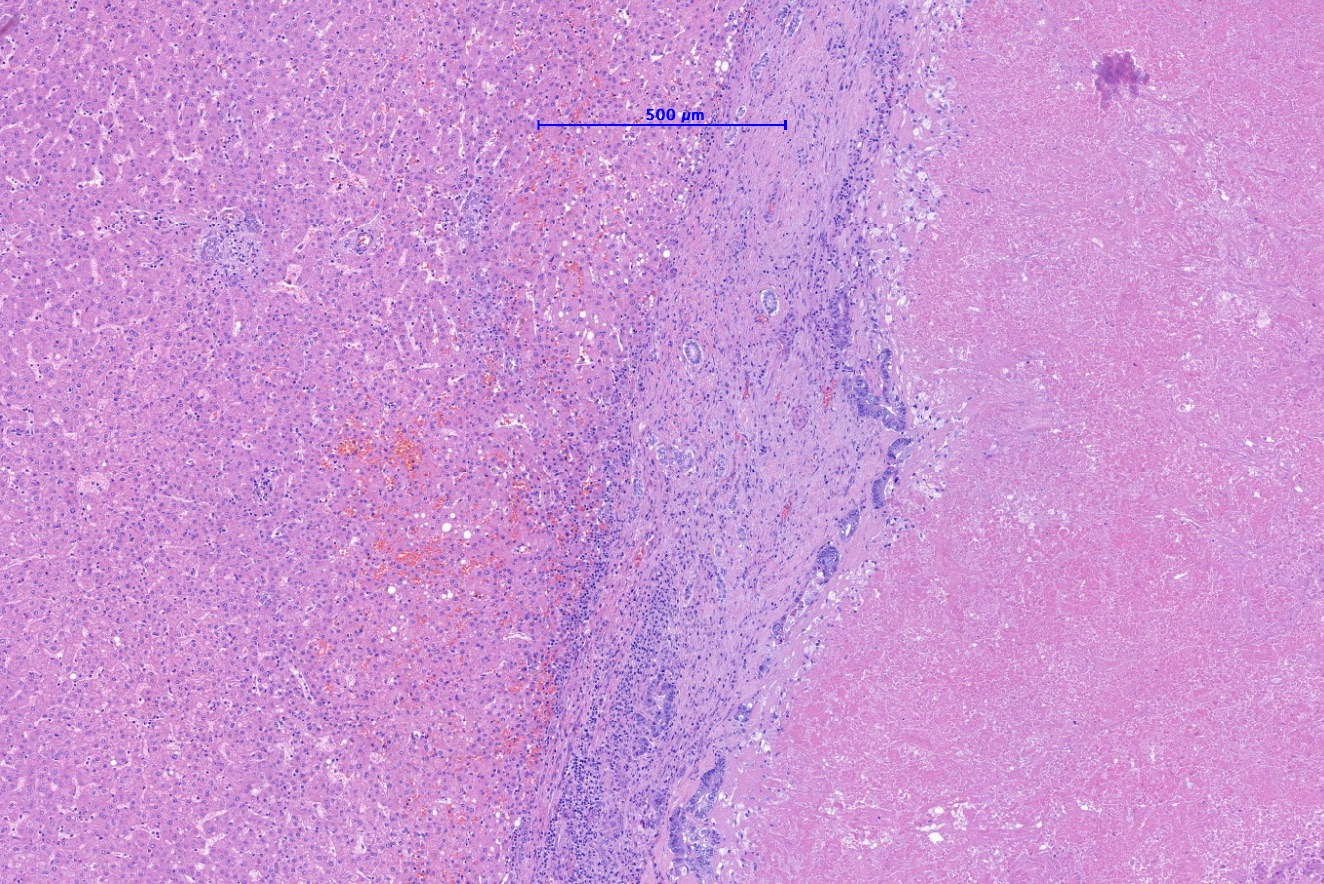


Supplementary Figure S2. Replacement HGP (cancer cells in contact with hepatocytes at interface between cancer and liver. No rim of fibrous tissue to separate cancer from liver).


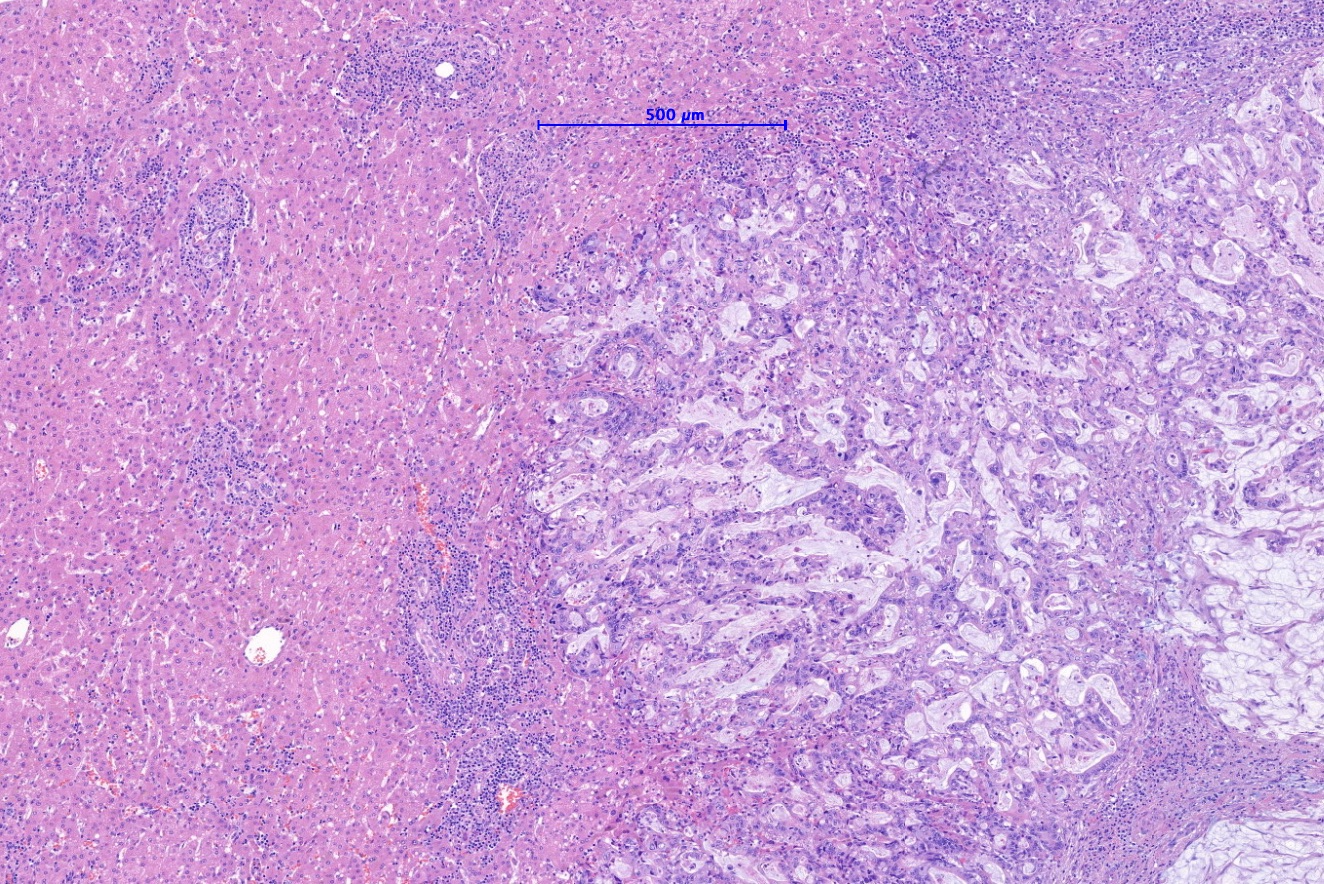


Supplementary Figure S3. Inflamed immune phenotype.


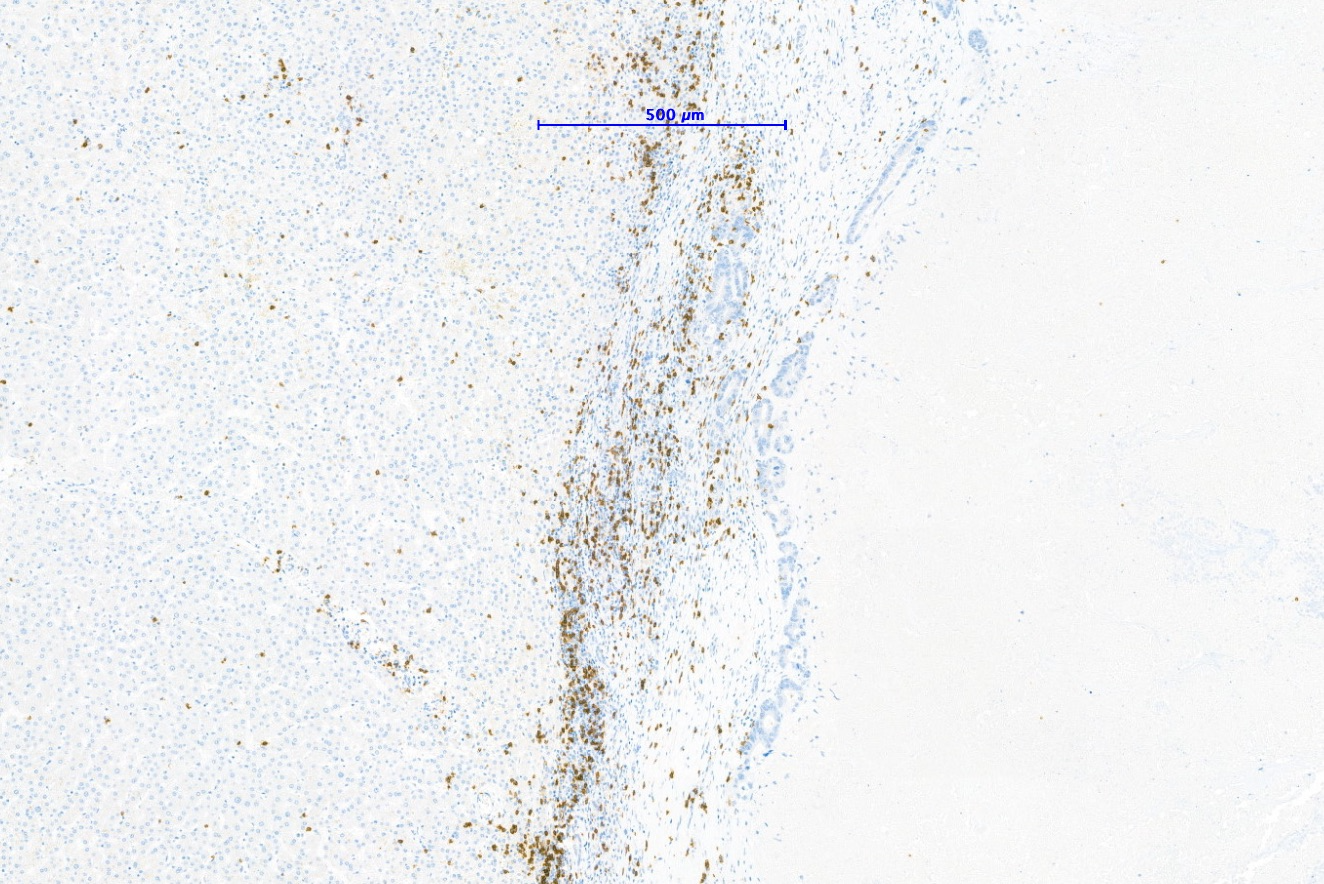


Supplementary Figure S4. Non-inflamed immune phenotype.


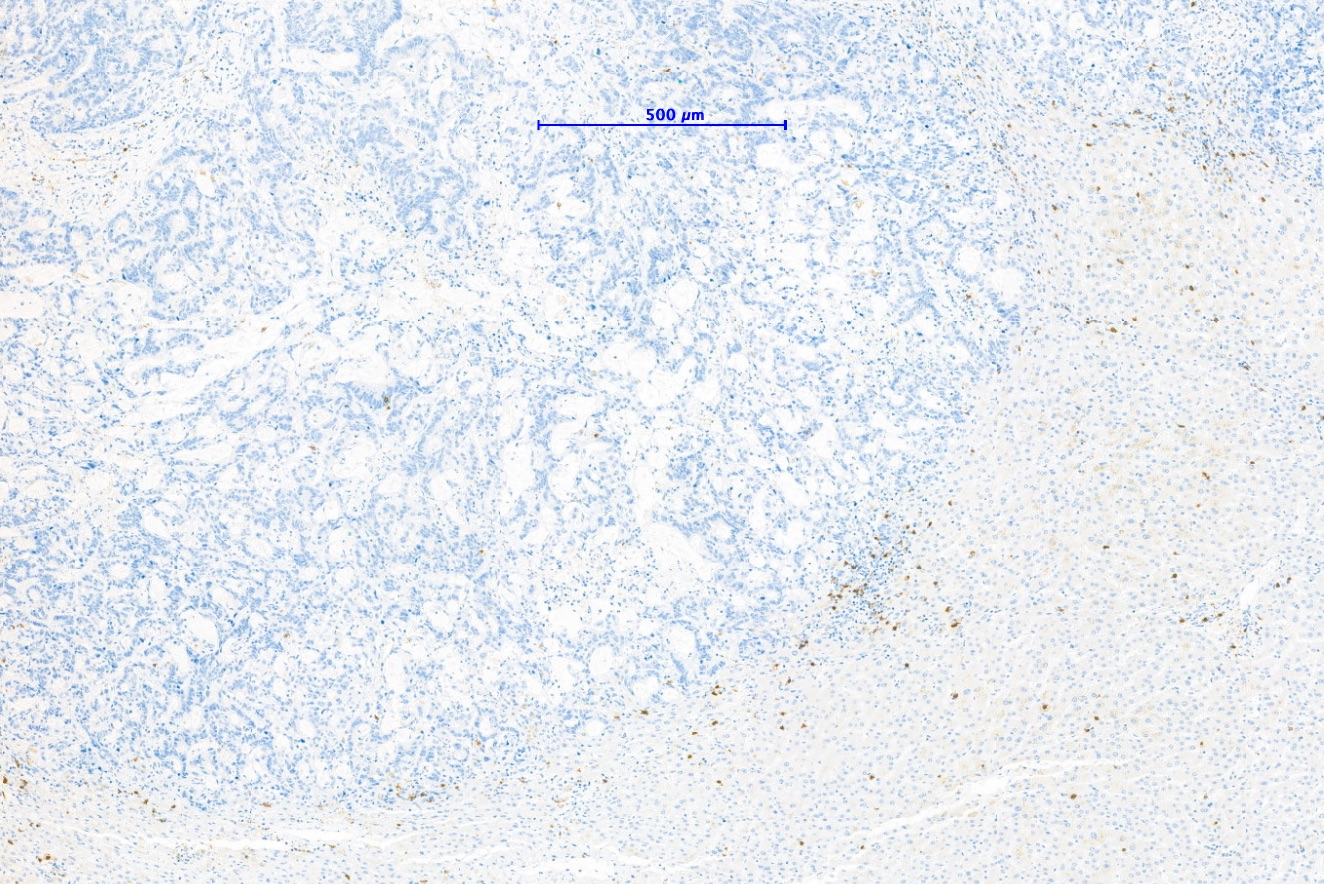

Supplement: Supplementary file 1 — Supplementary Files [file 41416_2020_812_MOESM1_ESM.doc]
